# An Evaluation Model of English Normal Students' Informatization Teaching Ability Based on Technical Pedagogical Content Knowledge and Few-Shot Learning

**DOI:** 10.1155/2022/8591303

**Published:** 2022-07-05

**Authors:** Honghong Zeng, Ronghua Tan

**Affiliations:** Yuzhang Normal University, Nanchang 330103, China

## Abstract

Information-based instruction is the most important and recently altered sort of instruction. Numerous changes in educational techniques have emerged from the ongoing growth of science and technology. Teachers must also grasp and enhance their capacity to educate with information on a regular basis. The goal of this research is to look at how to use the TPACK model and computational intelligence to build and explain a model for evaluating English normal students' informatization teaching skills. To measure information-based teaching competence, this research recommends using the Technical Pedagogical Content Knowledge model and Few-Shot Learning technology. As a result, this paper covers the principles and related algorithms of both, as well as constructing and assessing the case design and analysis of the information-based teaching ability evaluation model. According to the experimental data, the average CK of normal students' subject material knowledge is 3.522, which is the highest among the seven dimensions. The subject content is well-understood by ordinary students. When compared with teaching abilities, the competence level is low, and the performance of the integration of technology and teaching content needs to be improved.

## 1. Introduction

The rapid advancement of information technology has altered the way we work, learn, and play, and this is true in education as well. Education faces significant hurdles in today's quickly evolving digital culture. To raise the level of teacher education, a scientific and effective evaluation plan for teachers' information teaching competence is required. The TPACK framework is a knowledge system that combines technology into topic teaching in the classroom. Teachers actively select appropriate technical tools based on the actual teaching situation. It is the process of bringing individual topic expertise to teaching practice, with a focus on the importance of incorporating technology into the classroom.

It examines concerns linked to the cultivation of ordinary students' information teaching capacity using the TPACK model. This can provide a specific theory for nurturing ordinary students' information teaching ability, as well as a deeper understanding of teachers' information teaching ability from the theoretical standpoint of TPACK. TPACK opens up a new study field for the effective integration of information technology and curriculum, as well as theoretical direction for teachers on how to teach in a technological context more effectively. It is a novel idea to improve teacher professional development, and it is the knowledge base for teachers to effectively carry out instruction when the technology of the information age has invaded education.

The innovation of this paper is (1) through the combination of the TPACK model and information teaching, this paper introduces the theory and related methods of TPACK and computational intelligence in detail, as well as artificial neural network and genetic algorithm. (2) Based on a comprehensive review of the importance of information technology teaching ability, this paper proposes an index system for evaluating teachers' information technology teaching skills. And this paper uses the TPACK theoretical model to determine the structural relationship between the index system and teaching ability. Through weight calculation, it is concluded that the teaching ability level of normal students is relatively high, but the ability related to technology is relatively low, and the ability to integrate technology and subject content is not strong.

## 2. Related Work

With the development of information technology and the continuous advancement of educational informatization, TPACK has become an important standard for measuring teachers' teaching ability in the information age. The main purpose of Gomez-Trigueros I M is to evaluate the realization of spatial and digital competence through the intervention of the TPACK teaching model in the classrooms of primary school teachers at the University of Alicante. However, he did not take into account the influence of other factors [1]. Salas-Rueda RA analyzed the impact of the TPACK model in the design of predicate logic instructional units, taking into account the use of Raptor software, YouTube videos, and the social network Facebook. However, the number of samples in his experiments is small [2]. Almenara J C aimed to assess knowledge based on the TPACK (Technical, Pedagogical, and Content Knowledge) model and he used quantitative, descriptive, and relational methods. The conclusions he drew underscore the need for a kind of teacher training that is based not only on the technical aspects but also on the pedagogical and content aspects in an integrated approach. However, his views are not supported by a large amount of objective evidence [3]. D Mourlam used a mixed methods approach to attempt to describe teacher development experiences based on TPACK and adult learning theories directed by teacher developers. The findings suggest that teachers find the ongoing nature of the development experience valuable and value the support of teacher developers in achieving their goals. The survey results also showed an increase in TPACK for both teachers and teacher education candidates participating in the program. However, his experiments are one-sided [4]. Miguel-Revilla D analyzed the practical utility of the TPACK model and the effectiveness of a pedagogical intervention for social studies secondary education prospective teachers in a university setting over a two-year period. Finally, his experimental results can lead to the conclusion that the pedagogical and conceptual orientation of the teaching program has shown positive effects, demonstrating that the effectiveness of an integrated approach that can adapt to the specificities and challenges of social studies education. However, he ignored the interference of realistic factors [5]. Eutsler L explored how the use of TPACK's pedagogical knowledge structure and steps to release responsibility framework can help senior teachers design instruction to read and write using the iPad. His analysis revealed that teacher-controlled teaching methods attract the attention of experienced teachers to lesson plans. The pedagogical approach “teacher as facilitator” increases user confidence and exploration. Problem-based teaching methods help target individualized students. However, his analysis is not universal [6]. Stefani S aimed to describe the use of a Problem-Based Learning (PBL) model based on Theology, Teaching and Content Knowledge (TPACK) to improve the integrated topic learning process in V SDN 07 Pandam Gadang. The findings suggest that a Problem-Based Learning (PBL) model based on Technology, Pedagogical, and Content Knowledge (TPACK) can improve the integrated topic learning process in the elementary classroom. However, his model is not very feasible [7]. Starting from a theoretical framework, Bustamante C described the development of joint displays in mixed methods research case studies. However, the method he describes is somewhat monolithic [8].

## 3. TPACK and Computational Intelligence Methods

### 3.1. TPACK

#### 3.1.1. Connotation of TPACK

TPACK is a new concept emerging in the process of teacher specialization, and it is a comprehensive science and technology of knowledge teaching. It was originally called PACK but was changed to TPACK for ease of memory and pronunciation. TPACK provides a new way of thinking. Once it is proposed, it has attracted great attention of educational scientists at home and abroad. TPACK is a new requirement for teachers' computer skills.

If teachers can effectively integrate technology into their professional knowledge structure in a modern educational environment and complete teaching efficiently, it will also become an important criterion for measuring teachers' teaching ability in the information age.

#### 3.1.2. TPACK Structure

**Figure 1:**
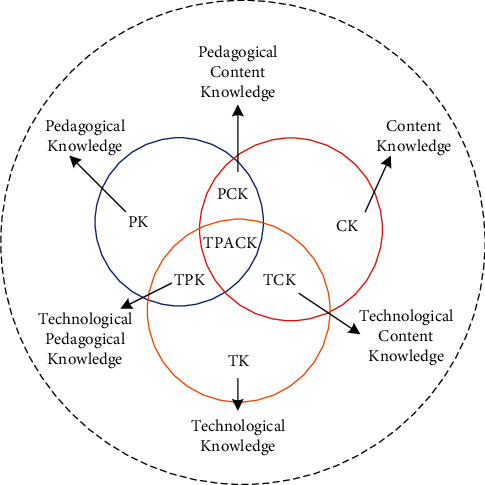
TPACK framework.

The use of instructional technology in educational contexts has become synonymous with technology integration. To help measure and guide teachers' use of educational technology, a variety of educational technology models and frameworks have been established. Many educational technology specialists believe that topic knowledge has an impact on how technology is used in the classroom. There are substantial contrasts, for example, between technology in science curriculum, successful curriculum integration, and effective technology and curriculum integration in social sciences. A new theory integrating pedagogical knowledge, subject knowledge, and technical knowledge (PK, CK, and TK) is shown in Figure 1.

This theoretical framework is called Technical Educational Content Knowledge (TPCK or TPACK) and expands its definition on the basis of the PCK knowledge structure, adding an element of technical support. The structural framework of TPACK consists of seven components, namely, TK, CK, PK, TPK, PCK, TCK, and TPACK.

1. *TK (Technological Knowledge).* Technical knowledge such as the use of blogs and various mobile devices, computers, projectors, and laboratory equipment is used in teaching. Office software such as PPT and word in teaching design is the necessary technical knowledge for teachers.
2. *PK (Pedagogical Knowledge).* It is general pedagogical knowledge. General teaching strategies include teaching of theorems, concepts, definitions, and arrangement of teaching links such as the design of lesson plans. It is the basic teaching method that teachers must master.
3. *CK (Content Knowledge).*It is subject knowledge required to be taught to students in various disciplines, such as laws, definitions, concepts, and formula theorems in science teaching. Students mastering these subject knowledge is the main goal of teaching but also the knowledge background that teachers must have.
4. *TCK (Technological Content Knowledge)*. It is disciplinary knowledge that integrates technology. Technical knowledge is used to help present a particular subject knowledge, but this composite knowledge is independent of pedagogical knowledge. For example, in the teaching of functions in mathematics courses, using technology to show the changing trend of functions can be considered as TCK.
5. *TPK (Technological Pedagogical Knowledge)*. It is pedagogical knowledge that integrates technology. The process of using technology to help teaching in the teaching of various disciplines is a combination of technology and pedagogical knowledge. This integrated pedagogical knowledge is not so much specific to the teaching of a specific subject as it is applicable to all subject pedagogical knowledge. The classroom discussion teaching is used in the Internet environment, that is, the learning environment supported by the computer (CSCL teaching environment).
6. *PCK (Pedagogical Content Knowledge).* It is subject teaching method knowledge. This knowledge is intended to enable students to experience a complete teaching experience so that they can understand and master the knowledge of the subject.
7. *TPACK (Technological Pedagogical Content Knowledge)*. There is a debate on the definition of TPACK. One is that TPACK is an extension of PCK; that is, the element of technology is added to Schulman's PCK theory. The other is that it is a cross combination of three basic elements (TK, CK, and PK), TPACK is to strengthen students in learning a certain subject, such as using Google Earth in geography teaching to help students explore and learn El Niño phenomenon. It is a fusion of technology and pedagogical knowledge of subject knowledge, designed to allow students to experience a certain learning process and better understand the content that needs to be mastered.

### 3.2. Computational Intelligence Technology

Computational intelligence methods [9, 10] are inspired by biological evolutionary mechanisms and some natural phenomena, and many new solutions to complex optimization problems are widely used in various fields of problems, concerns, and applications due to their high optimization performance and less requirement for specific information. Computational intelligence methods include neural network [11, 12], fuzzy control, genetic algorithm, immune computing, and swarm intelligence.

#### 3.2.1. Artificial Neural Network

**Figure 2:**
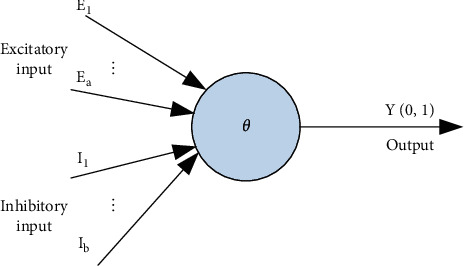
M-P model.

The artificial neural network [13, 14] is based on the physiological research results of brain tissue, simulates some mechanisms and mechanisms of the brain, starts from the basic functions of neurons, and gradually forms a network from simple to complex. Its organization simulates the interaction of biological nervous systems with real-world objects. It simulates parallel processing on a large scale, has strong robustness and fault tolerance, and has strong self-learning ability. The first one is about the most basic and primitive model in the neuron synapse model: the binary threshold unit (Binarythresholdunit), the famous M-P model, as shown in Figure 2.

In Figure 2, *θ* represents the threshold when neurons are excited, and the M-P model reflects the structural and functional characteristics of biological neurons to a certain extent. Its mathematical description is as follows:

(1) $$\begin{array}{c} \left\{\begin{array}{cc} \sum_{i=1}^{a}E_{i}-\theta \geq 0, & \text{and}\sum_{j=0}^{b}I_{i}=0,Y=1,\\ \text{other,} & \text{Y}=\text{0.} \end{array}\right) \end{array}$$

With proper setting of neuron thresholds and combination weights, the model can approximate any binary function and can complete any finite logical function. The basic idea is that when two neurons are excited or inhibited at the same time, the connection strength between them increases. It is mathematically expressed as follows:

(2) $$\begin{array}{c} \left\{\begin{array}{cc} \sum_{k=1}^{a}x_{i}^{\left(k\right)}x_{j}^{\left(k\right)}, & i\neq j,\\ 0, & i=j. \end{array}\right) \end{array}$$

The meaning of this learning rule is that the adjustment of the connection weight is proportional to the product of the activity states of the two neurons, and the connection weight is symmetric. The connection weight of a neuron to itself is zero, which is still used in various neural network models.

**Figure 3:**
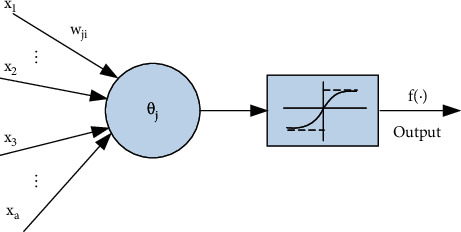
Neuron structure model.

A typical single artificial neuron model is shown in Figure 3.

The neuron input-output relationship is as follows:

(3) $$\begin{array}{c} y_{j}=f\left(\sum_{i=1}^{a}w_{ji}x_{i}-\theta_{j}\right)\\ =f\left(\sum_{a=1}^{a}w_{ji}x_{i}\right),\\ {\text{inx}}_{\text{0}}=\theta_{j},\\ w_{0}=-1. \end{array}$$

In the formula, the threshold is *θ*_*j*_, the link weight coefficient is *w*_*ji*_, and f(·) is the output transformation function, also known as the excitation function and the transfer function.

**Figure 4:**
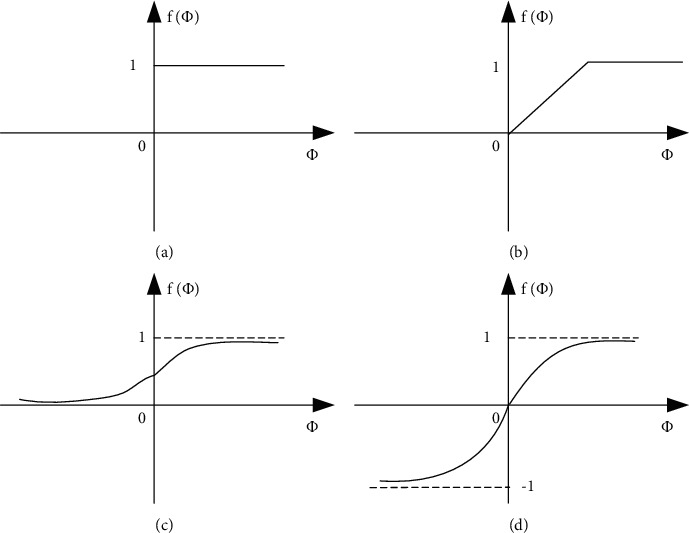
Neuron state transition function.

**Figure 5:**
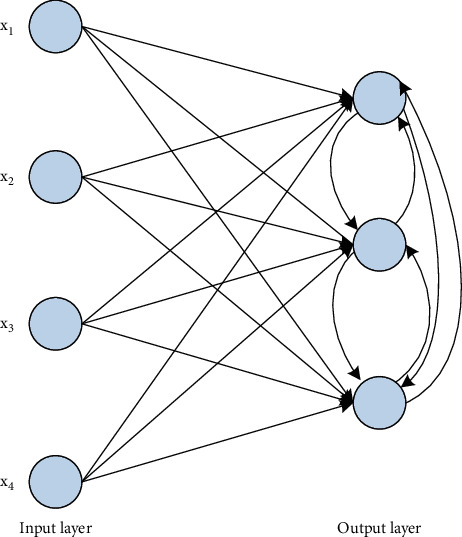
Competitive learning network with lateral inhibitory connections.

**Figure 6:**
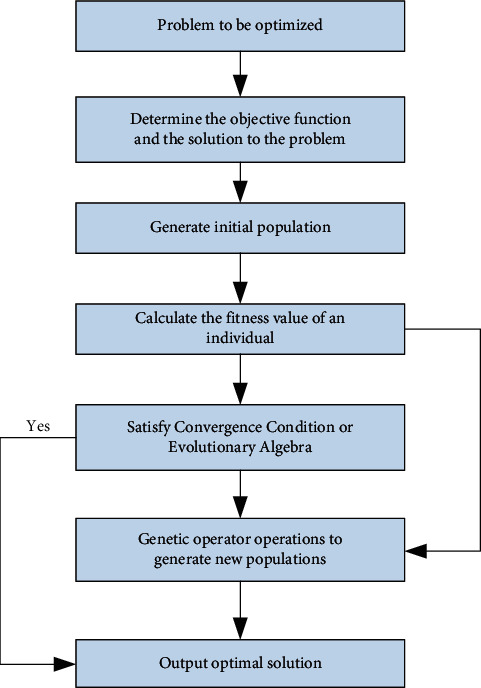
Basic flow chart of the simple genetic algorithm.

**Figure 7:**
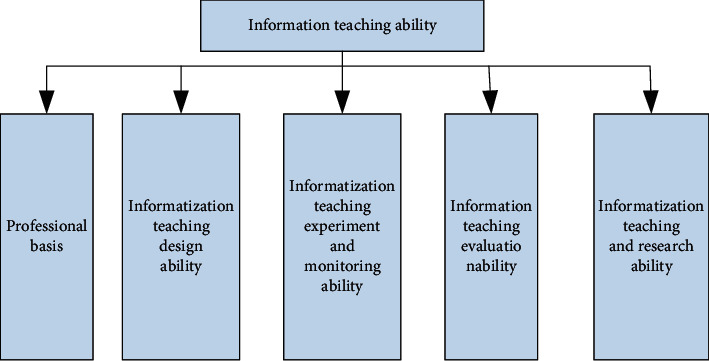
Theoretical framework of the first-level index of informatization teaching ability.

The more commonly used transfer functions are as follows:

(1) *Symbol Function*. As shown in Figure 4(a),
  (4) $$\begin{array}{c} y=f\left(\varphi \right)=\left\{\begin{array}{cc} 1, & \varphi \geq 0,\\ 0, & \varphi <0. \end{array}\right) \end{array}$$
(2) *Piecewise Linear Function*. As shown in Figure 4(b),
  (5) $$\begin{array}{c} y=f\left(\varphi \right)=\left\{\begin{array}{cc} 1, & \varphi \geq \alpha ,\\ \vartheta , & 0\leq \varphi <\alpha ,\\ 0, & \varphi <0. \end{array}\right) \end{array}$$
(3) *Sigmoid Function*. As shown in Figure 4(c),
  (6) $$\begin{array}{c} f\left(\varphi \right)=\frac{1}{1+e^{-\varphi }}. \end{array}$$
(4) *Hyperbolic Tangent Function*. As shown in Figure 4(d),
  (7) $$\begin{array}{c} f\left(\varphi \right)=\frac{1-e^{-\varphi }}{1+e^{-\varphi }}. \end{array}$$

Commonly used neural network learning algorithms are as follows:

*(1) Error Correction Learning*. *y*_*k*_(*t*) is the actual output of neuron k at time *t* when *x*_*k*_(*t*) is input, and *y*_*k*  *d*_(*t*) is the expected output, and then the error signal is as follows:

(8) $$\begin{array}{c} e_{k}\left(t\right)=y_{k\;d}\left(t\right)-y_{k}\left(t\right). \end{array}$$

The ultimate goal of error correction learning is to minimize a specific objective function *e*_*k*_(*t*), so that the actual output of each output unit on the network approximates the expected output in a statistical sense. The most commonly used objective functions are as follows:

(9) $$\begin{array}{c} \lambda \left(t\right)=\frac{1}{2}\sum_{k}e_{k}^{2}\left(t\right). \end{array}$$

Calculate the minimum value of *λ*(t) for the weight w as follows:

(10) $$\begin{array}{c} \Delta w_{kj}=\sigma e_{k}\left(t\right)x_{j}\left(t\right). \end{array}$$

In the formula, the learning step size is *σ*.

*(2) Hebb Learning*. “When neurons at both ends of a synapse are activated (activated and inhibited) at the same time, the strength of the connection should be strengthened, otherwise it should be weakened” is a summary of Hebb's learning rule. The mathematical expression is as follows:

(11) $$\begin{array}{c} \Delta w_{kj}\left(t\right)=F\left(y_{k}\left(t\right),x_{j}\left(t\right)\right). \end{array}$$

Among them, the states of neurons at both ends of *w*_*kj*_ are *y*_*k*_(*t*) and *x*_*k*_(*t*), and the most common cases are as follows:

(12) $$\begin{array}{c} \Delta w_{kj}=\sigma y_{k}\left(t\right)x_{j}\left(t\right). \end{array}$$

Since Δ*w*_*kj*_ is proportional to the correlation between *y*_*k*_(*t*) and *x*_*j*_(*t*), it is sometimes called the correlation learning rule.

*(3) Competitive Learning*. In the competitive learning process, each network output unit competes with each other and finally only one strongest unit is activated, as shown in Figure 5. The most common case is inhibitory lateral phase connections between output neurons. So, in the initial output unit, if one unit is stronger, it will win and overwhelm the others, and in the end, only this stronger unit will be active.

The most commonly used competitive learning rules can be written as follows:

(13) $$\begin{array}{c} \Delta w_{kj}=\left\{\begin{array}{cc} \sigma \left(x_{i}-w_{ji}\right), & \text{Ifneuron}j\text{competestowin,}\\ 0, & \text{Ifneuron}j\text{failstocompete.} \end{array}\right) \end{array}$$

#### 3.2.2. Genetic Algorithm

Genetic algorithm is an optimization method that simulates natural selection and genetic mechanisms, simulating the phenomena of reproduction, hybridization, and mutation in natural selection and natural genetic processes. When solving a problem using a genetic algorithm, each possible solution to the problem is encoded as a “chromosome,” that is, an individual and thousands of individuals form a group (all possible solutions). At the beginning of a genetic algorithm, some individuals (i.e., initial solutions) are always randomly generated. Each individual is evaluated against a predetermined objective function, which gives a fitness value. Based on this fitness value, it selects individuals to replicate in the next generation. The selection operation embodies the principle of “survival of the fittest,” where “good” individuals are selected for replication and “bad” individuals are eliminated. Then, the selected individuals are recombined through crossover and mutation operators to generate a new generation. Because this group of new individuals inherits some excellent characters of the previous generation, their performance is better than that of the previous generation, which gradually evolves towards a better solution. Therefore, the genetic algorithm can be regarded as a process of gradual evolution of a group composed of feasible solutions. The basic process of the genetic algorithm is shown in Figure 6.

The execution process of the genetic algorithm contains many random operations, so it is necessary to analyze its mathematical mechanism. A schema is a collection of strings that take the same characters at certain positions. Extract the following symbols as follows:

  P: a certain mode
 
*f*_*i*_: fitness of the i-th string (solution)
 
$\bar{f}\left(t\right)$: the average fitness of the t-th generation population
 
$\bar{f}\left(P,t\right)$: the average fitness of pattern P in the t-th generation population
 
*n*_*i*_(*P*, *t*+1): the expected value of the number of offspring produced by the solution *i* of the P mode of the *t* generation in the *t* + 1 generation
 
*n*(*P*, *t*): the number of solutions belonging to pattern P in the t-th generation
 
*φ*(*P*): the definition distance of P
 
*O*(*P*): the order of P

Consider first the outcome of the selection. In the standard genetic algorithm, the selection criteria are based on the principle of proportionality; therefore, through the action of the ith selector, the expected value of the number of people who will continue to exist in the next generation is *n*(*f*_*i*_/∑*f*) and then

(14) $$\begin{array}{c} \bar{f}\left(P,t\right)=\frac{1}{n\left(P,t\right)}\sum f_{i}. \end{array}$$

Then,

(15) $$\begin{array}{c} n\left(P,t+1\right)=n\left(P,t\right)\cdot \bar{f}\frac{\left(P,t\right)}{f\left(t\right)}. \end{array}$$

The formula shows that the role of the selection operator will increase (decrease) the ability of a pattern that is above (below) average in its applicability across generations, improving quality.

This plan can obviously be maintained in the next generation if there is no intersection or if the intersection point is beyond the character positions specified on the left and right ends of Figure 6. Therefore, the probability *R*_*s*_ that the P mode will continue to exist in the next generation should satisfy

(16) $$\begin{array}{c} R_{s}\geq 1-R_{c}\cdot \frac{\varphi \left(P\right)}{\left(L-1\right)}. \end{array}$$

Taking into account the effects of selection and crossover, there are

(17) $$\begin{array}{c} n\left(P,t+1\right)\geq n\left(P,t\right)\cdot \bar{f}\left(P,t\right)\cdot \left[1-R_{c}\cdot \frac{\varphi \left(P\right)}{\left(L-1\right)/\bar{f}\left(t\right)}\right). \end{array}$$

Finally, because *R*_*m*_ represents the probability of the mutation operator acting, the constant probability is 1 − *R*_*m*_.

(18) $$\begin{array}{c} {\left(1-R_{m}\right)}^{O\left(P\right)}\approx 1-R_{m}\cdot O\left(P\right). \end{array}$$

The probability of unreserved is about *O*(*P*) · *R*_*m*_. Therefore, considering the functions of selection, crossover, and mutation operators, we end up with

(19) $$\begin{array}{c} n\left(P,t+1\right)\geq n\left(P,t\right)\cdot \bar{f}\left(P,t\right)\cdot \frac{\left[1-R_{c}\cdot \varphi \left(P\right)/L-1\cdot R_{m}\right]}{\bar{f}\left(t\right)}. \end{array}$$

This result is the so-called model theorem. If only the influence of the selection operator is considered, the amount of solution contained in the standard solution increases or decreases with the passage of generations, which is related to the average suitability of the standard solution. Specifically, if $\bar{f}\left(P,t\right)=f\left(t\right)\left(1+c\right)$, *c* > 0 is a constant and then

(20) $$\begin{array}{c} n\left(P,t\right)=n\left(P,t-1\right)\left(1+c\right)=n\left(P,O\right){\left(1+c\right)}^{t}. \end{array}$$

That is, functions with high average fitness grow exponentially in their ability to compete with other functions. However, high average conditions alone are not enough to guarantee high growth rates. When considering other impacts in detail, the definition of an operating model that requires good quality should be smaller in length and shorter in scope. High average suitability modes, low resolutions, and low order distances enable exponential growth in the number of solutions contained in generation after generation of group transmissions, which is the essence of the model theorem [15].

### 3.3. Information-Based Teaching Ability

“Competence” is the subjective condition that people need to solve problems, complete specific tasks, and be competent. Informatization teaching ability refers to the ability of teachers to rationalize and optimize the teaching process under the condition of providing sufficient information to use information technology and information resources. It is a reflection of the comprehensive quality of teachers combining various technologies and resources to carry out practical teaching. It is a composite of various abilities that teachers have in the integration of information technology and curriculum, and it points to the level of teaching practice, the application of technology, and the ability to integrate [16, 17].

In this study, the training object of information-based teaching ability is normal students. In view of the fact that normal students are in the survival stage of teachers' professional development, they mainly focus on whether they can stand on the podium and how to complete a class but do not really pay attention to students. In addition, the main task of normal students is to learn the theoretical knowledge related to the professional development of teachers and to imitate the theoretical knowledge learned into practice; in this process, there are fewer opportunities to actually contact students. During the four-year study process, the normal students in most normal colleges and universities in China are limited by various conditions, and the contact time with students ranges from 2 to 4 months.

The research on information-based teaching ability emerges along with the research on teachers' ability, which is the product of the information age. Awareness of technology integration, subject teaching methods, and knowledge are important ways to improve teachers' information teaching ability and teaching ability. Teachers' computer skills are the ability to implement TPACK. In other words, teachers' informatization teaching ability is their ability to integrate technology, classroom pedagogy, and subject content knowledge.  Figure 7 shows the content related to the first-level indicators of information-based teaching ability.

**Figure 8:**
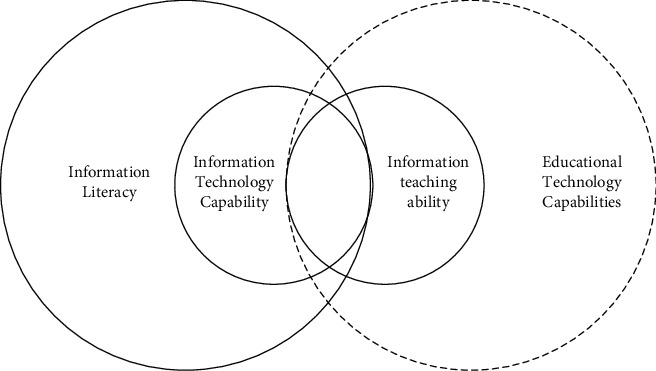
The relationship between information teaching ability and related concepts.

Informatization teaching ability and educational technology ability are included, and the relationship between information literacy and information technology ability is a cross relationship, with the same part and different parts [18]. The relationship between the four is shown in Figure 8.

## 4. Experiment and Analysis on the Evaluation Model of Normal Students' Informatization Teaching Ability

### 4.1. Investigation and Analysis of TPACK Level of English Normal Students

The scale is a common tool for measuring the level of TPACK. In order to ensure the scientificity and validity of the scale, the accuracy of the data, and the objectivity of the results, the questionnaire was tested in this study. The test subjects were English normal students from normal universities, a total of 100 people, and 100 questionnaires were distributed, all of which were valid questionnaires.

The questionnaire is divided into two parts, one part is the TPACK level survey scale for normal students and the other part includes 7 basic information questions and 2 open-ended questions. In order to test the scientificity and accuracy of the questionnaire design, the author uses SPSS19 software to test the reliability and validity of the scale, so as to make the questionnaire more reasonable and effective. Reliability refers to the stability, reliability, and consistency of the results presented by the measurement tools. The higher the reliability is, the more stable and reliable the measurement results are. If the *a* coefficient of the questionnaire is between [0.7, 0.8], it means that the reliability of the questionnaire is high, and the *a* coefficient between [0.8∼0.9) means that the reliability of the questionnaire is very high. A coefficient above 0.9 indicates that the reliability of the entire questionnaire is very high.

The internal consistency of each part of the TPACK scale was tested by Cronbach's alpha using SPSS19 software, and the results are shown in Table 1.

It can be seen from the reliability analysis of the questionnaire in Table 1 that, in each dimension of the scale, except for the PCK dimension, the reliability of the remaining dimensions is above 0.8. It can be seen that the internal consistency of the scale is relatively satisfactory, and the overall reliability is high, which can be used for research.

Validity testing usually includes two parts, content validity and construct validity, which are tested after many discussions and revisions. Therefore, it ensures the scientific nature of the questionnaire content validity test to a certain extent. In the structural validity test of the scale, factor analysis is usually used to verify it according to the KMO decision criteria. The value of KMO is between 0 and 1. The larger the KMO value, the more common factors among the variables in the questionnaire and the more suitable for factor analysis. When the KMO is less than 0.5, it means that it is not suitable for factor analysis, and when the KMO is greater than 0.8, it means that the quantity is expressed to a good degree [19].

The construct validity of the TPACK scale was tested using SPSS19 software, and the results are shown in Tables 2 and 3.

It can be seen from the reliability analysis of the questionnaire in Table 2 that the KMO values of each category of the scale are all above 0.70. The overall KMO was 0.819, which was greater than 0.8 in the Kaiser decision criterion, the chi-square value of Bartlett's sphericity test was 1633.041, the degree of freedom P was 528, and the Sig. was 0.000, reaching a significant level (*P* < 0.05). Based on the data analysis [20], the scale has common factors in the overall correlation table. According to its construct validity analysis, the correlation factors among different dimensions of the questionnaire and the correlation between dimensions, the coefficients reached a significant level, indicating that the scale has high construct validity. Overall, the TPACK Normal Student Research Scale has high reliability and validity.

**Figure 9:**
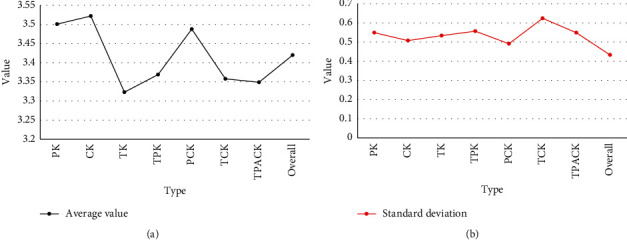
Descriptive analysis of each dimension of TPACK.

In order to investigate the overall level of TPACK of normal students, English normal students from normal universities were selected as the research objects. In order to reflect the overall situation of TPACK, this paper selects a total of 100 normal students in different stages from freshman to junior year and distributes 100 questionnaires, all of which are valid questionnaires. In order to understand the overall level of TPACK of normal students, this paper uses SPSS19 software to analyze the mean and standard deviation of the survey results by dimension, as shown in Figure 9.

It can be seen from Figure 9 that the average value of each TPACK dimension of normal students is *M* = 3.420, which is between “average” and “good,” which is a medium level. Second, there are differences between the dimensions of TPACK for normal students. The seven dimensions present different levels, and they are sorted to obtain TK < TPACK < TCK < TPK < PCK < PK < CK. The highest among the seven dimensions is knowledge of course content, with an average CK of 3.522, indicating that normal students have higher knowledge of subject content than other dimensions. The average values of the three dimensions of PCK's subject pedagogy knowledge, PK's pedagogical knowledge, and CK's subject content knowledge are also higher. It shows that normal students have high self-confidence in subject content knowledge and pedagogical knowledge.

### 4.2. Scenario Application Ability of Informatization Teaching

**Figure 10:**
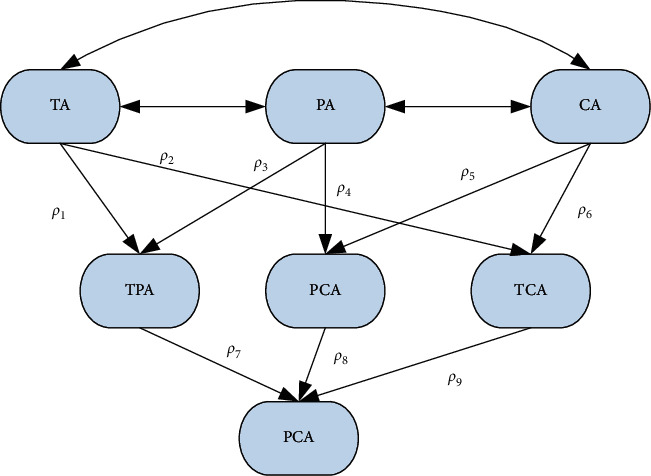
The structure model of the evaluation index of information teaching ability.

According to the research results of the TPACK structure model and the specific meaning of the indicators for evaluating information teaching ability, Figure 10 shows the structure model of the evaluation index of normal students' informatization teaching ability.

In terms of the quantitative relationship between the evaluation indicators of informatization teaching ability, the path coefficients in the model are *ρ*1∼*ρ*6. They represent the influence weights of the three second-level indicators of the basic ability of informatization teaching on the three second-level indicators of the integration and application ability of informatization teaching. Among them, *ρ*1 and *ρ*2 are the influence weights of technology-related capabilities on the ability to integrate technology and teaching and the ability to integrate technology and subject content, respectively. *ρ*3 and *ρ*4 are the influence weights of teaching-related ability on integration of technology and teaching ability and subject teaching ability, respectively. *ρ*5 and *ρ*6 are the influence weights of subject-related ability on subject teaching ability and ability to integrate technology and subject content, respectively. The path coefficients *ρ*7∼*ρ*9, respectively, represent the influence weights of the three secondary indicators of the integrated application ability of informatization teaching on the situational application ability of informatization teaching. Among them, *ρ*7 is the influence weight of integrating technology and teaching ability on the subject teaching ability integrating technology. *ρ*8 is the influence weight of subject teaching ability on subject teaching ability integrating technology. *ρ*9 is the influence weight of integrating technology and subject content ability on the subject teaching ability integrating technology.

Regarding the calculation of the weight, the ability to apply the IT teaching status has the most important influence on the teacher's informatization teaching ability. The weight of the secondary indicator integrated technology subject teaching ability is defined in 1.00. The weight should be calculated according to the index influence path coefficient reflected in the teaching ability rating index structure model. Based on this information, the influence path coefficient of this paper on the subject teaching ability of integrated technology is shown in Table 4.

### 4.3. Evaluation Model of Informatization Teaching Ability

**Figure 11:**
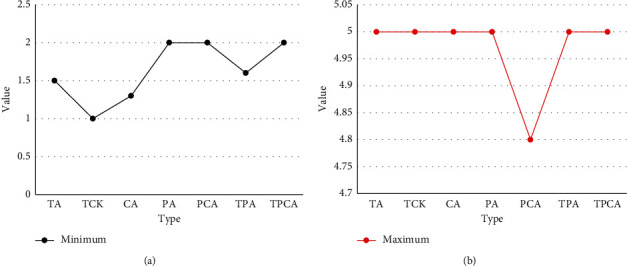
The minimum value and maximum value of the evaluation index of information teaching ability.

**Figure 12:**
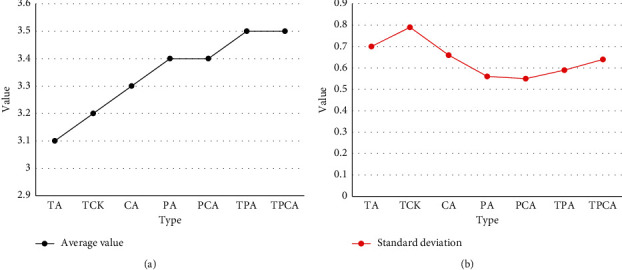
Mean and standard deviation of the evaluation indicators of information-based teaching ability.

In order to verify the validity of the teacher's structured information evaluation model and calculate the weight evaluation of each index, this study carried out an empirical research on the evaluation of information-based teaching ability. The seven subindicators should be measured proportionally, and the measurement data should be modeled through a path analysis model to determine the weight of each indicator. The empirical research results show that the actual measurement data are in good agreement with the structural model of the evaluation index of information teaching ability. This also shows that the model can more accurately evaluate the informatization teaching ability of normal students. According to the weight model of indicators at all levels, among the three first-level indicators, the ability to apply educational information context is crucial. The ability to integrate and apply information teaching is secondary, and the basic skills of informatics teaching are the least important. Among all secondary indicators, the target teaching ability index of integrated technology has the largest weight, the integrated technology and teaching ability index has the second weight, and the ability index related to subject teaching has the lowest weight. Figures 11 and 12 are the descriptive statistical analysis results of each index level and the overall level of informatization teaching ability.

After completing the three-year teacher training course, the general level of information teaching ability of normal students is relatively high. Among the three first-level indicators, the ability to implement the state of informatization teaching is the highest, the completion and application index of computer teaching is at the middle level, and the basic skills index of computer teaching [21] is at the middle level. Among the seven secondary evaluation indicators, the skill index related to technology is the lowest, and the skill index of teaching objects related to technology is the highest [22]. Among the three secondary indicators of ability to complete and implement computer-based instruction, the level of ability to integrate technology and subject content was lower than the other two indicators. Among the three secondary indexes of basic computer teaching ability, the level of technology-related ability indexes is lower than the other two indexes.

To sum up, normal students have high information teaching ability, which to a certain extent reflects that general education has achieved remarkable results in cultivating teachers' information teaching ability. The measurement results show that normal students have high teaching ability and can well integrate technology, teaching methods, and content to form good teaching decisions. Compared with teaching ability, normal students have relatively low technology-related abilities, and their ability to integrate technology and disciplinary content is poor. In the future, the proportion of courses related to technology integration in different subject courses should be increased to promote the comprehensive development of normal students' information teaching ability, thereby improving the level of educational development.

## 5. Discussion

First of all, through the study of relevant knowledge points of literature works, this paper initially masters the relevant basic knowledge and analyzes how to conduct research on the evaluation of the information-based teaching ability of medical-oriented English normal students based on the TPACK model and computational intelligence. This paper expounds TPACK and informatization teaching ability, focuses on the method of computational intelligence, and analyzes the evaluation of informatization teaching ability based on TPACK and computational intelligence through experiments. And this paper constructs the evaluation model of information teaching ability. This paper also focuses on artificial neural network and genetic algorithm, and artificial neural network is an artificial neural network created according to the physiological structure and information processing process of the brain, imitating human intelligence. The genetic algorithm is more suitable for large scale, large population, complex environment, and unclear problem structure [23, 24].

Through experimental analysis, this paper shows that normal students have high informatization information teaching ability and teaching ability and can well integrate technology, teaching methods, and content to form good teaching decisions. Compared with teaching ability, normal students have lower skill levels and lower performance in integrating technology and teaching content.

## 6. Conclusion

Computer science is an inevitable trend of future development, and computer science teaching is an important part of future education development. The final foothold of the evaluation index system of teachers' informatization teaching ability is still practice. This research also hopes to update the teaching concept ideologically and improve teachers' understanding of the development of information-based teaching. Teachers should actively use multimedia technology to assist teaching, optimize the teaching process, fundamentally change the traditional teaching structure and teaching methods, and achieve the goal of quality education in the new era. Although the traditional exam-oriented education is strong, the development of science and technology is rapid. With the popularization of computer teaching methods, teachers' computer teaching ability has been continuously improved. With the implementation of the quality education policy, the overall quality of national education will surely rise to a higher level.

## Figures and Tables

**Table 1:** Questionnaire reliability analysis table.

Dimension	Number of items	Cronbach's alpha coefficient	Cronbach's alpha coefficients based on standardized terms
PK	7	0.89	0.89
CK	4	0.81	0.81
TK	8	0.81	0.81
TPK	6	0.88	0.88
PCK	5	0.72	0.72
TCK	4	0.81	0.82
TPACK	6	0.88	0.88
Overall	40	0.95	0.95

**Table 2:** KMO and Bartlett sphericity test.

Kaiser–Meyer–Olkin measure of sampling adequacy		0.819
Bartlett's sphericity test	Sig.	0.000
	Approximate chi-square	1633.041
	df	526

**Table 3:** Questionnaire construct validity analysis.

Dimension	PK	CK	TK	TPK	PCK	TCK	TPACK	Overall
PK	1							
CK	0.615^*∗∗*^	1						
TK	0.396^*∗∗*^	0.614^*∗∗*^	1					
TPK	0.574^*∗∗*^	0.659^*∗∗*^	0.598^*∗∗*^	1				
PCK	0.666^*∗∗*^	0.580^*∗∗*^	0.463^*∗∗*^	0.608^*∗∗*^	1			
TCK	0.409^*∗∗*^	0.573^*∗∗*^	0.530^*∗∗*^	0.676^*∗∗*^	0.608^*∗∗*^	1		
TPACK	0.678	0.539^*∗∗*^	0.534^*∗∗*^	0.789^*∗∗*^	0.677^*∗∗*^	0.721^*∗∗*^	1	
Overall	0.720^*∗∗*^	0.768^*∗∗*^	0.732^*∗∗*^	0.880^*∗∗*^	0.824^*∗∗*^	0.821^*∗∗*^	0.884^*∗∗*^	1

**Table 4:** Weights of evaluation indicators of information-based teaching ability.

First-level indicator	Index weight	Secondary indicators	Index weight
Basic ability of informatization teaching	*ρ*_*TA*_+*ρ*_*PA*_+*ρ*_*CA*_/*ρ*_SUM_	TA	*ρ*_*TA*_=*ρ*_1_ × *ρ*_7_+*ρ*_2_ × *ρ*_9_
		PA	*ρ*_*PA*_=*ρ*_3_ × *ρ*_7_+*ρ*_4_ × *ρ*_8_
		CA	*ρ*_*CA*_=*ρ*_5_ × *ρ*_8_+*ρ*_6_ × *ρ*_9_

Informatization teaching integration application ability	*ρ*_*TCA*_+*ρ*_*PCA*_+*ρ*_*TCA*_/*ρ*_SUM_	TPA	*ρ*_*TPA*_=*ρ*_7_
		PCA	*ρ*_*PCA*_=*ρ*_8_
		TCA	*ρ*_*TCA*_=*ρ*_9_
Informatization teaching situation application ability	*ρ*_TPCA_/*ρ*_SUM_	TPACK	*ρ*_TPCA_/*ρ*_SUM_=1.00

## Data Availability

The data used to support the findings of this study are available from the corresponding author upon request.
